# Sodium Salt of Partially Carboxymethylated Sodium Alginate-g-Poly(acrylonitrile): I. Photo-Induced Synthesis, Characterization, and Alkaline Hydrolysis

**DOI:** 10.3390/gels9050410

**Published:** 2023-05-15

**Authors:** Jignesh Trivedi, Arvind Chourasia

**Affiliations:** 1 Post Graduate Department of Chemistry, Sardar Patel University, Vallabh Vidyanagar 388120, India; 2 Tridev Resins (India) Pvt. Ltd., 136/E-1, II Phase, G.I.D.C., Vapi 396195, India

**Keywords:** photo-induced grafting, acrylonitrile, sodium salt of partially carboxymethylated sodium alginate, optimal reaction conditions, saponification, characterization

## Abstract

An efficient redox initiating system, ceric ammonium nitrate/nitric acid, has been employed for the first time to carry out photo-induced graft copolymerization of acrylonitrile (AN) onto sodium salt of partially carboxymethylated sodium alginate, having an average degree of substitution value to be 1.10. The photo-grafting reaction conditions for maximum grafting have been systematically optimized by varying the reaction variables such as reaction time, temperature, the concentration of acrylonitrile monomer, ceric ammonium nitrate, and nitric acid, as well as the amount of the backbone. The optimum reaction conditions are obtained with a reaction time of 4 h, reaction temperature of 30 °C, acrylonitrile monomer concentration of 0.152 mol/L, initiator concentration of 5 × 10^−3^ mol/L, nitric acid concentration of 0.20 mol/L, amount of backbone of 0.20 (dry basis) and the total volume of the reaction system of 150 mL. The highest percentage of grafting (%G) and grafting efficiency (%GE) achieved are 316.53% and 99.31%, respectively. The optimally prepared graft copolymer, sodium salt of partially carboxymethylated sodium alginate-g-polyacrylonitrile (%G = 316.53), has been hydrolyzed in an alkaline medium (0.7N NaOH, 90–95 °C for ~2.5 h) to obtain the superabsorbent hydrogel, H–Na–PCMSA–g–PAN. The chemical structure, thermal characteristics, and morphology of the products have also been studied.

## 1. Introduction

Chemical modification of biopolymers via graft copolymerization is the most attractive method because it helps in modifying the chemical and physical properties of biopolymers [1,2,3], thereby enlarging the range of its utilization. This investigation has been primarily taken to justify the premise that graft copolymerization is a very important technique to customize the properties of biopolymers. The synthesis of graft copolymers based on biopolymers involves the use of chemical and radiation (high- and low-energy) methods. However, the photo-grafting method has several advantages [4,5,6]: less degradation of the backbone polymer, and more control over the grafting reaction, in addition to achieving higher grafting efficiency, lower operation cost, and UV light absorption.

Sodium alginate (SA), an industrially important marine biopolymer derived mainly from brown algae, consists of two monomeric units, β-D-mannuronic acid (M) and α-L-guluronic acid (G) [7]. SA has a wide range of applications, such as the controlled release of drugs [8], pesticides [9], as a thickener, gelling agent and colloidal stabilizer [10]. Thus, although this important biopolymer, SA, is widely used in many fields despite beingprone to enzymatic degradation, it suffers from limitations in fabrication, which limit its use in fields such as pharmaceuticals and medicine. To overcome this problem, graft copolymerization of different vinyl monomers onto SA has been successfully carried out using various methods [11,12,13,14,15,16,17,18,19,20,21,22]. However, a comprehensive literature survey revealed that apart from our previously reported study [23], no report has been published on the case of photo-grafting of various vinyl monomers onto sodium alginate as well as its derivatives, including carboxymethylated ones.

In this research, SA was first modified via carboxymethylation followed byphoto-grafting of acrylonitrile (AN) onto sodium salt of partially carboxymethylated sodium alginate (Na–PCMSA, $\bar{\mathrm{DS}}$= 1.10) using cerium ammonium nitrate (CAN) as a photo-initiator. The use of AN as a grafted monomer in this present work is due to its excellent grafting efficiency [24], the improvement of heat resistance of the graft copolymer [25], and also the subsequent alkaline hydrolysis of the graft copolymer to obtain a water absorbent [4]. The photo-grafting reaction was optimized by systematically studying the effect of various reaction variables on the grafting yields. The results are discussed. The structure and properties of the products were characterized via FTIR, SEM, and TGA.

The optimally synthesized graft copolymer, Na–PCMSA–g–PAN (%G = 316.53,%GE = 99.31) was finally saponified to prepare a novel superabsorbent hydrogel, H–Na–PCMSA–g–PAN, which exhibited very high capability of water absorption in low-conductivity water as well as the different saline solutions. The swelling behavior of the hydrogel in various swelling media has been studied, and water absorption results are analyzed with a view to exploring the studies of the swelling kinetics of the hydrogel [26].

## 2. Results and Discussion

### 2.1. Synthesis of Na-PCMSA-g-PAN

**Figure 1:**
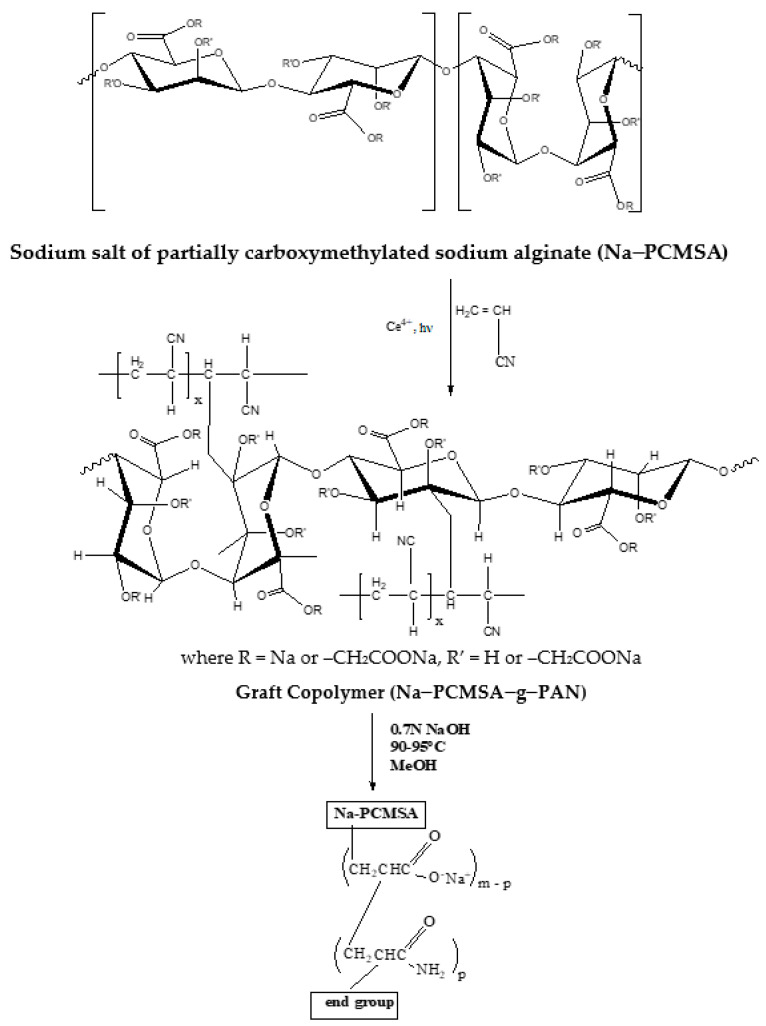
The synthetic route for the photo-graft copolymer, Na–PCMSA–g–PAN, and its saponification for the formation of the superabsorbent hydrogel, H–Na–PCMSA–g–PAN.

In the present case of Ce(IV)–initiated photo-graft copolymerization, the oxidation reaction of Ce(IV) with Na-PCMSA will occur preferably at the C_2_-C_3_ glycol unit and to a lesser extent at the C_6_ primary hydroxyl as a result of one electron process [27]. The photo-initiator, Ce(IV) ion initially forms a Ce(IV)-Na-PCMSA complex, which is then reduced to Ce(III) ion with the formation of free radicals at either C_2_ or C_3_ on the trunk polymer. The proposed mechanism pathway for the synthesis of the graft copolymer, Na–PCMSA–g–PAN, has been depicted in Figure 1.

### 2.2. Influence of the Polymerization Variables on the Grafting Yields

#### 2.2.1. Calculation of Grafting Yields

The values of the grafting yields, viz. the percentage of grafting (%G),percentage of grafting efficiency (%GE), and percentage of homopolymer (%Hp), were evaluated with the help of the following expressions [28]:

(1) $$(i)\;\%G\;=\;\frac{Wt.\;of\;polymer\;grafted}{Initial\;wt.\;of\;backbone\;}\;\times \;10^{2}$$

(2) $$(ii)\;\%GE\;=\;\frac{Wt.\;of\;polymer\;grafted}{Wt.\;of\;polymer\;grafted\;+\;Wt.\;of\;homopolymer\;}\;\times \;10^{2}$$

(3) $$(iii)\;\%\;Homopolymer\;(\%H_{p})\;=\;100-\%GE$$

#### 2.2.2. Influence of Polymerization Variables

With a view to obtaining the optimal conditions for photo-grafting copolymerization, the effects of certain variables on polymerization were studied systematically. The results regarding the influence of the reaction variables on the grafting yields (%G, %GE, and %Hp) are discussed below.

##### The Effect of Na-PCMSA Amount

**Figure 2:**
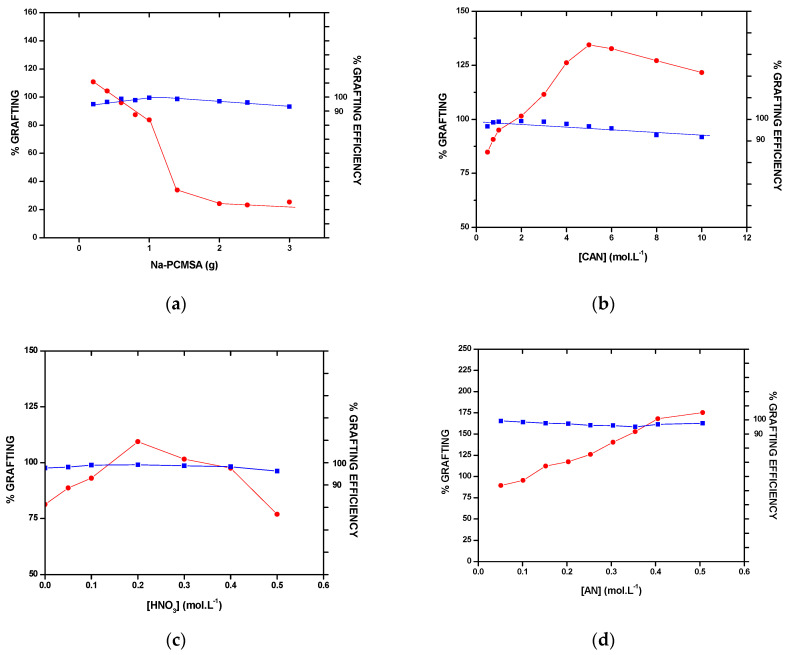

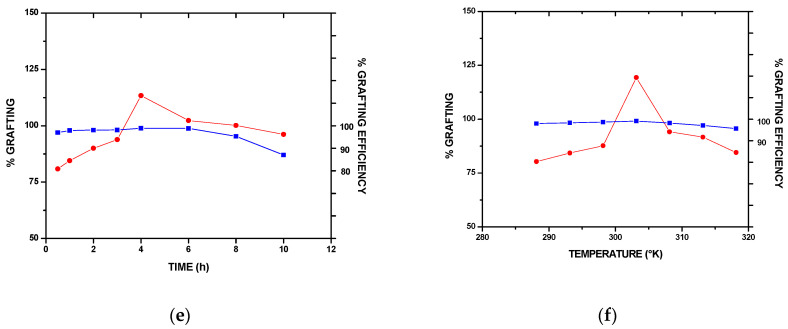
Influence of (**a**) amount of sodium salt of partially carboxymethylated sodium alginate (Na–PCMSA); (**b**) ceric ammonium nitrate (CAN) concentration; (**c**) nitric acid (HNO_3_) concentration; (**d**) acrylonitrile (AN) concentration; (**e**) reaction time and (**f**) reaction temperature on -●- %G or -■- %GE.

The effect of the amount of Na–PCMSA (0.2 g–3.0 g) on the grafting yields (%G and %GE) is shown in Figure 2a. It is evident from the figure that the value of %G decreases with an increasing amount of Na–PCMSA. However, the value of %GE increases very slowly in the beginning up to 1.0 g. of Na–PCMSA; thereafter, it decreases with increased amounts of Na–PCMSA. The continuous decrease in %G is traced to the fact that with an increasing amount of concentration of Na–PCMSA, the weight of the grafted PAN chains will increase, resulting in a decrease in the monomer-to-backbone ratio. Besides increasing the amount of Na–PCMSA, the formation of a greater number of macroradicals takes place, thereby making the termination rate of photo-graft copolymerization faster than the rate of initiation, as a result of which %GE also decreases. In the literature, similar outcomes have previously been documented [12,28,29].

##### The Photo-Initiator Concentration

The concentration of CAN was varied in the range (0.50 × 10^−3^ mol/L to 10 × 10^−3^ mol/L) to study its effect on grafting yields. The results are depicted in Figure 2b. The maximum value of %G (134.43%) was achieved at [Ce^4+^] = 5 × 10^−3^ mol/L, where homopolymer content was only 3.22%. Increased photo-initiator concentration resulted in the formation of more radical sites on the Na–PCMSA backbone that, in turn, led to higher %G and lower homopolymer formation. However, because the CAN photo-initiator was used as a dilute solution in HNO_3_, at CAN concentration higher than 5 × 10^−3^ mol/L, a more acidic pH probably partially terminates the macroradicals on the Na–PCMSA. As a result, the decrease in the values of grafting yields with a photo-initiator concentration beyond its optimum value (5 × 10^−3^ mol/L) is observed [cf. Figure 2b]. In the literature [30,31,32], similar observations have also been made and published.

##### The Acid (HNO_3_) Concentration

The concentration of HNO_3_ was varied from 0.051 to 0.506 mol/L to investigate its effect on grafting yields, and the results are presented in Figure 2c. Initially, the values of the grafting yields are found to be increased with the increase in nitric acid concentration up to 0.20 M. This is attributed to the increase in the concentration of [Ce^4+^] and [Ce(OH)_3_]^3+^ at the expense of [Ce-O-Ce]^6+^. The size of [Ce]^4+^ and [Ce(OH)_3_]^3+^ ions is smaller than that of [Ce-O-Ce]^6+^ ion, and, therefore, they are more effective in their ability to form complexes with the Na–PCMSA backbone than [Ce-O-Ce]^6+^ ion. Beyond [Ce]^4+^ = 0.20 M, it is observed that grafting yields decrease. This can be explained due to the fact that as the nitric acid concentration increases, the formation of more and more [Ce]^3+^ and [Ce(OH)_3_]^3+^ takes place, which in turn affects grafting yields adversely. In the literature [33,34,35], similar outcomes are described.

##### The Monomer Concentration

Monomer concentration affects the grafting yields. Accordingly, the monomer (AN) concentration varied from 0.051 to 0.506 mol/L when all the other conditions were kept constant. The results obtained are shown in Figure 2d. It is observed from Figure 2d, the value of %G increased gradually with the monomer concentration up to 0.405 mol/L, and then it became constant with a further increase in the monomer concentration. As the concentration of monomer increases, the diffusion of the monomer molecules into the backbone increases, resulting in a higher yield of grafting. However, the value of %GE decreases steadily with increasing the monomer concentration [cf. Figure 2d]; that may be attributed to the increase in the concentration of PAN macroradicals and thereby the rates of combination and disproportionation of PAN and monomer become faster than the rate of their combination with the Na–PCMSA molecules. This will result in the formation of a homopolymer; thereby, the viscosity of the medium increases, and the monomer diffusion into the Na–PCMSA backbone becomes more difficult, leading to a decrease in %GE. Similar behaviors were also seen in the previous research [11,16,30,33,36].

##### The Reaction Time

The influence of photo-polymerization time on the yields of grafting was studied by varying the reaction time from 0.5 h to 10 h keeping other variables such as temperature, amount of backbone, and concentrations of monomer, photo-initiator, and nitric acid constant. The results of this experiment are depicted in Figure 2e. It is seen from the figure that %G increased progressively with an increase in reaction time up to 4 h, reaching a maximum value of %G to be 113.35%; the value of the percent grafting efficiency (%GE) also increased very slowly, and the highest value obtained was 98.87% at 4 h. 

The increase in grafting yields is attributed to the increase in the number of grafting sites on the backbone, and more monomer molecules will add to the growing grafted chains. After the optimum reaction time (4 h), the decrease in the values of %G and %GE is due to the decreased number of available active free radical sites for photo-grafting and retardation of diffusion of reactants. In addition, the longer UV irradiation time has a detrimental effect on the grafted chains of PAN in the presence of a photo-initiator which is responsible for the decrease in the grafting yield values. The literature has also documented a comparable reliance on grafting yields [28,31,32,34,37].

##### The Reaction Temperature

The influence of temperature [Figure 2f] on the grafting yields (%G and %GE) was studied at different temperatures ranging from 15 to 45 °C. It is seen from the figure that the value of %G increased with the rise of temperature from 15 to 30 °C and then with a further increase in temperature it decreased. The value of %GE also behaved in a similar fashion. The maximum values of the grafting yields, %G = 119.31 and %GE = 99.13 were obtained at 30 °C. The increase in %G up to 30 °C could be attributed to the increased rate of photolysis of the Na–PCMSA-Ceric complex so that more active sites are generated on the Na–PCMSA backbone resulting in the increased propagation of thephoto-graftcopolymerization onto Na–PCMSA. The enhanced diffusion of monomer (AN) and photo-initiator (CAN) into and onto Na–PCMSA backbone and the increased mobility of the monomer molecules and their higher collision probability with the backbonemacroradicals also resulted in the increase in %G up to the said temperature. However, the favored chain termination reactions, chain transfer reactions, and increase in the formation of homopolymer resulted in a decrease in the grafting yield values beyond 30 °C. Other researchers have found similar results [30,31,32,38,39].

Thus, the optimal reaction conditions evaluated in the photo-initiated graft copolymerization were Na–PCMSA = 0.20 g (dry basis), [CAN] = 5 × 10^−3^ mol/L, [HNO_3_] = 0.20 mol/L, [AN] = 0.152 mol/L, Time = 4 h, Temperature = 30 °C and Total Volume = 150 mL. The highest values of %G = 316.53 and %GE = 99.31 were obtained under the optimum reaction conditions evaluated. 

### 2.3. Saponification or Alkaline Hydrolysis

The optimally synthesized PAN–grafted Na–PCMSA (%G = 316.53) was hydrolyzed under alkaline conditions (0.7N sodium hydroxide, 90–95 °C, ~2.5 h) by following the methanol precipitate method [40] for the formation of superabsorbent hydrogel, H–Na–PCMSA–g–PAN. The nitrile substituents of PAN were converted to a mixture of carboxamide, sodium carboxylate, and ammonia generated in the process (Figure 1). The reaction mixture initially assumed a deep orange-red color when reacted with alkali due to the formation from PAN of a highly conjugated polymer intermediate. The heterocycles are then opened via hydrolysis [to from hydrophilic carboxamide (–CONH_2_) and carboxylate (${CO}_{2}^{-}$) groups], continuing with a resulting color change from red to light yellow. This discoloration may be used as a practical indication to halt the alkaline treatment. As a result, the starting hydrophobic graft copolymer sample, Na–PCMSA–g–PAN, is converted to a hydrophilic gel, i.e., superabsorbent hydrogel (H–Na–PCMSA–g–PAN).

### 2.4. Characterization 

#### 2.4.1. FTIR Spectroscopy

**Figure 3:**
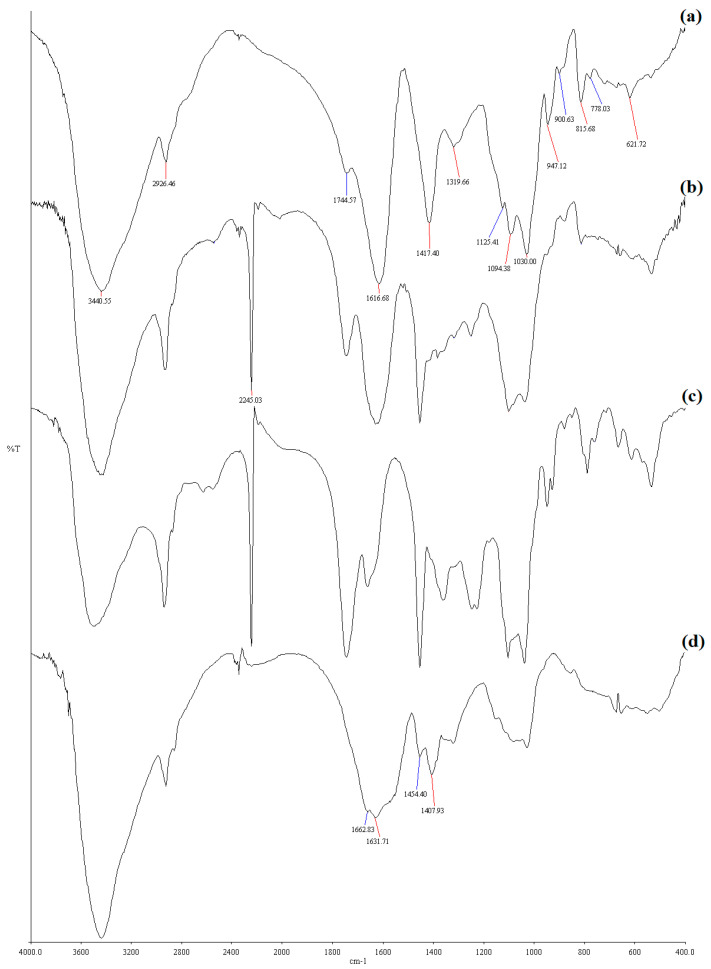
FTIR spectra of the following:(**a**) sodium salt of partially carboxymethylated sodium alginate (Na–PCMSA, ($\bar{\mathrm{DS}}$ = 1.10); (**b**) Na-PCMSA–g–PAN (%G = 316.53); (**c**) PAN; (**d**) the superabsorbent hydrogel, H–Na–PCMSA–g–PAN.

The structures of the graft copolymer and the superabsorbent hydrogel were confirmed from IR data. The IR spectrum of Na–PCMSA ($\bar{\mathrm{DS}}$ = 1.10) is shown in Figure 3a. The presence of a strong and broad absorption band at ~3440 cm^−1^ is assigned to O–H stretching vibrations. The absorptionbandat ~2926 cm^−1^ may be attributed to the –CH stretching. The C–O stretching is distributed in the absorptions at about 1125 cm^−1^, 1094 cm^−1^, and 1030 cm^−1^. The strong absorption at 1745 cm^−1^ is assigned to C=O stretching, suggesting the presence of –COO moiety in Na–PCMSA. The presence of the –COO moiety is evident from the absorption bands that appeared at 1617 cm^−1^ (due to asymmetric stretching of the moiety) and 1417 cm^−1^ (due to symmetric stretching of the moiety) [Figure 3a]. The IR spectra of Na–PCMSA–g–PAN and PAN (isolated by hydrolysis), respectively, are presented in Figure 3b,c. In addition to the absorptions of Na–PCMSA, an additional absorption band at ~2245 cm^−1^ showing the presence of –C≡N is seen in Figure 3b. The PAN isolated from the graft copolymer also showed an absorption at ~2245 cm^−1^ [Figure 3c]. Thus, the absorption in the IR indicated that the grafting occurred. 

The IR absorption of the hydrogel, H–Na–PCMSA–g–PAN, is indicated in Figure 3d. The IR absorption at ~1564 cm^−1^ and ~1407 cm^−1^ are attributed to asymmetric and symmetric stretching of the carboxylate moiety, respectively. The absorption at ~1454 cm^−1^ may have some contributions from the symmetric stretching mode of the said group. The peaks at ~1663 cm^−1^ and ~1632 cm^−1^ have contributions from C=O stretching vibrations coupled with NH_2_ bending and N-H bending, respectively. The strong absorption of the -C≡N group completely disappeared after hydrolysis; this may be regarded as the conversion of the nitrile groups into hydrophilic groups after the alkaline hydrolysis of the graft copolymer. The hydrophilic groups may be responsible for imparting super swelling behavior of the H–Na–PCMSA–g–PAN.

#### 2.4.2. Thermogravimetric Analysis (TGA)

**Figure 4:**
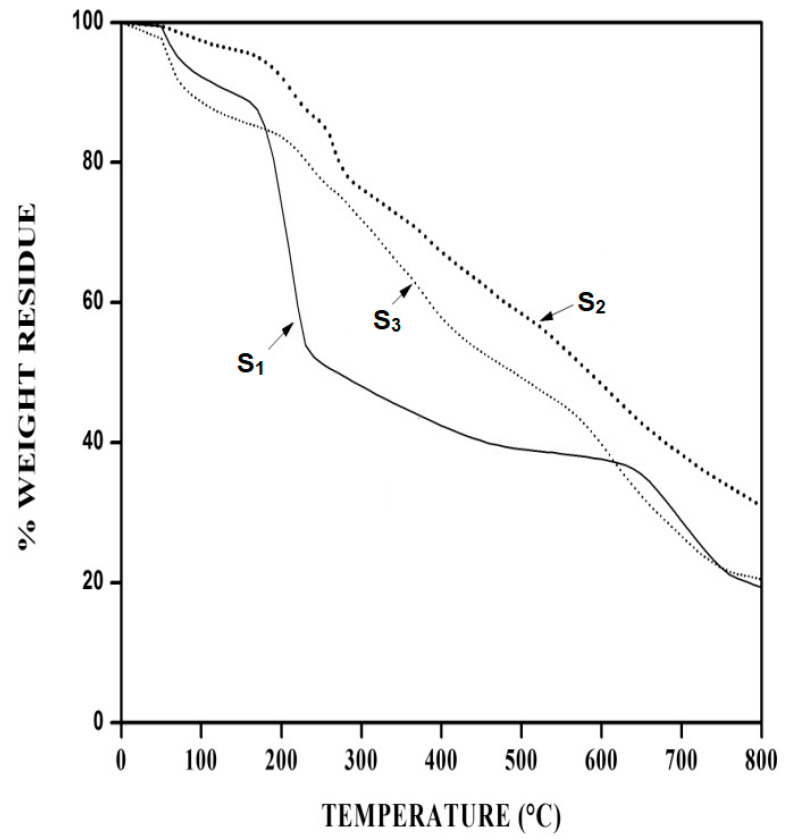
TG thermograms for (―) Na–PCMSA ($\bar{\mathrm{DS}}$ = 1.10 (S_1_); (•••) Na–PCMSA–g–PAN (%G = 316.53) (S_2_) and (…) H–Na–PCMSA–g–PAN (S_3_) at 10 °C/min. The insert shows the first derivatives of the curves shown in the figure.

The traces of TGA and DTG for Na–PCMSA (S_1_), Na–PCMSA–g–PAN (S_2_), and the superabsorbent hydrogel, H–Na–PCMSA–g–PAN (S_3_) samples are represented in Figure 4. Thermogravimetric data derived from these traces for all three samples are also tabulated in Table 1. It becomes evident from the TGA trace of Na–PCMSA (S_1_) that the decomposition process occurred in three stages. The one in the range of temperature 50–100 °C corresponds to the evaporation of water (7.19% weight loss). The major weight loss (40.64%) took place in the temperature range 135.95–280.85 °C (second step of decomposition). The DTG of Na–PCMSA also exhibited the temperature for the maximum decomposition for this stage at 212.88 °C. Beyond 280.85 °C, the sample degrades very slowly up to 552 °C, and, thereafter, it degrades with a maximum weight loss at 701.51 °C involving about 19.35% weight loss. The final decomposition temperature (FDT) was found to be 796.53 °C.

The graft copolymer, Na–PCMSA–g–PAN (S_2_), shows six-stage decomposition patterns. The grafting of acrylonitrile onto Na–PCMSA makes the graft copolymer, Na–PCMSA–g–PAN, hydrophobic. At the initial stage in the temperature range of 50–100 °C, the observed minor weight loss of about 2.09 wt% is ascribed to the loss of absorbed moisture, indicating that the graft copolymer Na–PCMSA–g–PAN is much more hydrophobic. On the other hand, the hydrogel H–Na–PCMSA–g–PAN (S_3_) shows a large amount of absorbed moisture (9.07% weight loss), which may be attributed to the presence of hydrophilic groups, such as $–{\mathrm{COO}}^{-}$ and –CONH_2_ in the hydrogel. The DTG curve of Na–PCMSA–g–PAN exhibited five steps in the temperature range of 66.88–136.13, 136.13–239.93, 239.93–317.77, 341.53–430.15, and 501.60–776.24 °C with the corresponding maximum temperatures at 92.82, 211.79, 270.26, 384.83, and 592.42 °C.

The superabsorbent hydrogel, H–Na–PCMSA–g–PAN (S_3_), shows a three-stage decomposition pattern in the temperature range 154.41–263.50, 263.50–469.99, and 530.65–782.64 °C involving about 9.33%, 24.19%, and 25.93% weight loss, respectively. The corresponding maximum decomposition temperatures occurred at 233.35, 378.25, and 611.79 °C, respectively. Upon comparing the thermal characteristics values (Table 1), such as T_20_ and char yield (C. Y.) at 800 °C for Na–PCMSA, Na–PCMSA–g–PAN, and H–Na–PCMSA–g–PAN samples, it becomes evident that the values of T_20_ and char yield at 800 °C are higher in the case of Na–PCMSA–g–PAN (T_20_ = 274.46 °C and C. Y. = 30.88%) in comparison with H–Na–PCMSA–g–PAN (T_20_ = 234.44 °C and C. Y. = 20.42%) and Na–PCMSA (T_20_ = 191 °C and C. Y. = 19.28%), indicating the following order of thermal stability:

Na–PCMSA–g–PAN >H–Na–PCMSA–g–PAN > Na–PCMSA.

Thus, the overall thermal stability of both grafted and hydrolyzed grafted copolymer is improved in comparison with Na–PCMSA itself. This may be attributed to the formation of conjugated cyclic systems consisting of -C=N- groups from the pyrolytic addition reaction of adjacent nitrile groups [41] in the case of Na–PCMSA–g–PAN. However, there is no nitrile group in the hydrogel H–Na–PCMSA–g–PAN structure, but the existence of COO^−^Na^+^, COOH, and CONH_2_ groups improve its thermal stability over Na–PCMSA.

#### 2.4.3. Scanning Electron Microscopy (SEM) Analysis

**Figure 5:**
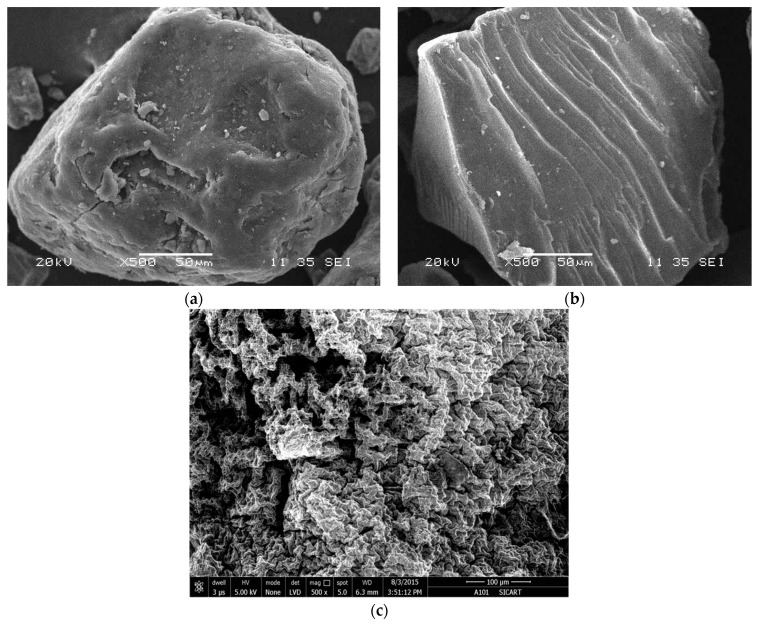
Scanning electron micrographs of (**a**) Na–PCMSA ($\bar{\mathrm{DS}}$ = 1.10) (500×), (**b**)Na–PCMSA–g–PAN (%G = 316.53) (500×), and (**c**) the superabsorbent hydrogel H–Na–PCMSA–g–PAN (500×).

The scanning electron micrographs obtained for Na–PCMSA ($\bar{\mathrm{DS}}$ = 1.10), Na–PCMSA–g–PAN (%G = 316.53), and the superabsorbent hydrogel, H–Na–PCMSA–g–PAN samples are represented in Figure 5a, 5b, and 5c, respectively. It appears from the SEM of the Na–PCMSA [Figure 5a] sample, that it has got smooth surface. However, upon grafting AN onto it, the morphology of the Na–PCMSA sample changed. Twisted filament-like morphology is observed [Figure 5b] due to the grafted hydrophobic PAN chains and/or general arrangement of the polysaccharide–g–PAN chains. The graft copolymer morphology [Figure 5b] was converted to a porous structure when it was treated in the alkaline medium to obtain H–Na–PCMSA–g–PAN [Figure 5c]. These pores are the regions of water permeation, and these are the sites of interaction with external stimuli.

## 3. Conclusions

This work is the first report to evaluate the optimum reaction conditions in the case of photo-grafting of AN onto Na–PCMSA ($\bar{\mathrm{DS}}$ = 1.10). The dependence of the grafting yields versus the reaction variables: time (0.5–10 h), reaction temperature (15–45 °C), concentrations of AN monomer (0.051–0.506 mol/L), CAN (0.5 × 10^−3^–10 × 10^−3^ mol/L), and HNO_3_ (Nil-0.5 mol/L), as well as the amount of Na–PCMSA (0.2–3.0 g, dry basis), was investigated systematically to optimize the photo-graft copolymerization. The maximum values of the grafting yields under the evaluated optimal reaction conditions were found to be %G = 316.53 and %GE = 99.31. FTIR spectrum of the graft copolymer, Na–PCMSA–g–PAN, confirmed the existence of a chemical link between the Na–PCMSA and PAN. The thermal stability of the graft copolymer was found to increase after being grafted with polyacrylonitrile, and scanning electron microscopy (SEM) micrographs revealed that the grafted and nongrafted Na–PCMSA samples were quite clearly different. 

The optimally prepared Na–PCMSA–g–PAN (%G = 316.53 and %GE = 99.31) was hydrolyzed in an alkaline medium, during which the nitrile groups of PAN were converted to a mixture of hydrophilic carboxamide and carboxylate groups followed by an in situ crosslinking of the grafted PAN chains leading to the formation of the superabsorbent hydrogel (H–Na–PCMSA–g–PAN) network with high water absorption capacity [26].

In the FTIR spectrum of the hydrogel, the disappearance of the nitrile sharp peak at ~2245 cm^−1^ and the appearance of the two distinct absorption bands at ~1564 cm^−1^ and~1407 cm^−1^ indicatethe respective presence of C=O asymmetric and symmetric stretching modes of the carboxylate anion, and the absorption bands appeared at ~1663 cm^−1^ and ~1632 cm^−1^, indicating contributions from C=O stretching vibrations coupled with NH_2_ bending and N-H bending, respectively, in carboxamide functional groups provided measure proofs for the conversion of the nitrile groups into carboxamide and carboxylate groups during alkaline hydrolysis of the graft copolymer. 

TGA analysis has proven that the overall thermal stability of the hydrogel was improved in comparison with Na–PCMSA ($\bar{\mathrm{DS}}$ = 1.10). SEM studies illustrated that the superabsorbent hydrogel (H–Na–PCMSA–g–PAN) had a loose and porous structure, facilitating the permeation of water into the polymer network.

Thus, as per the hypothesis stated earlier, the chemical and physical properties of the carboxymethylated derivative of sodium alginate (Na–PCMSA, $\bar{\mathrm{DS}}$ = 1.10), upon photo-induced grafting are found to be modified significantly. The synthesized superabsorbent hydrogel, H–Na–PCMSA–g–PAN, in the powder form, exhibited high water absorbency in low-conductivity water as well as different saline solutions, including simulated urine (SU), as discussed in part two of this communication [26], which may find potential application for personal health care products.

## 4. Materials and Methods

### 4.1. Materials

Loba Chemie Pvt. Ltd., Mumbai (India) supplied SA. The AN obtained from Fluka was distilled at atmospheric pressure and the middle fraction was collected and used.CAN was obtained from Qualigens, Glaxo India, India, and used as received. A fresh solution of CAN was prepared in analar grade nitric acid supplied by Qualigens, Glaxo India, India. All other reagents and solvents used were of reagent grade. Nitrogen gas was purified by passing through a fresh pyrogallol solution. Low-conductivity water was employed for the photo-graft copolymerization reactions. 

### 4.2. Methods

#### 4.2.1. Preparation of Sodium Salt of Partially Carboxymethylated Sodium Alginate (Na–PCMSA) 

As described earlier [33,42], sodium salt of partially carboxymethylated sodium alginate (Na–PCMSA) was prepared. The degree of substitution ($\bar{\mathrm{DS}}$) of Na–PCMSA was measured to be 1.10.

#### 4.2.2. Photo-Initiated Synthesis of Poly(acrylonitrile) Grafted Na–PCMSA (Na–PCMSA–g–PAN)

**Figure 6:**
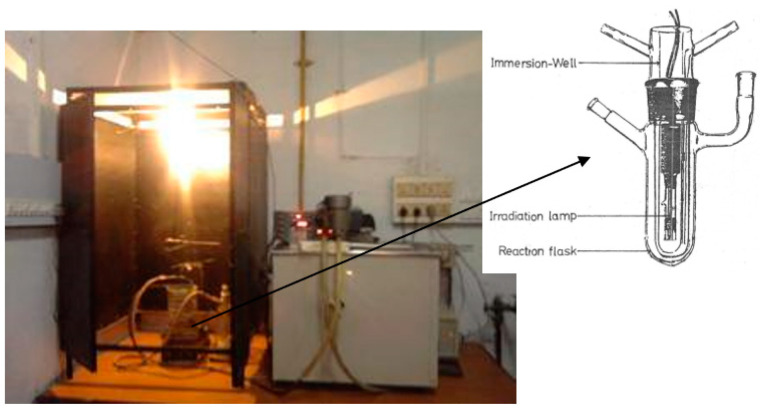
Detailed experimental setup for carrying out photo-graft copolymerization reaction.

Photo-chemical reactor supplied by Scientific Aids and Instruments Corporation (SAIC, Madras, India) was used to prepare the photo-induced graft copolymer Na–PCMSA–g–PAN [23]. A known amount of Na–PCMSA (0.2–3.0 g, dry basis) dissolved in low-conductivity water (in such a way that the total volume of the reaction system remains 150 mL), and the solution was stirred at 35 °C for an hour, followed by 20 min stirring at room temperature. Freshly prepared CAN solution (0.5 × 10^−3^–10.0 × 10^−3^ mol/L) in 10 mL nitric acid (Nil-0.5 mol/L) was added to the reaction flask, and contents were then flushed with purified nitrogen gas for half an hour, followed by an addition of known concentration of freshly distilled AN (0.051–0.506 mol/L). The reaction flask in the photo-chemical reactor was irradiated with a 125 W medium-pressure mercury lamp with a continuous flow of nitrogen gas and stirring for different time intervals (0.5–10 h) in the temperature range of 15–45 °C. After the stipulated time, the crude product was isolated by centrifugation and then purified by washing with dilute nitric acid as well as 95% methanol and finally washed with pure methanol. The crude copolymer of Na–PCMSA–g–PAN obtained was dried under vacuum at 40 °C. Figure 6 shows the photograph of the complete experimental setup for carrying out the photo-grafting reaction. 

#### 4.2.3. Purification of the Graft Copolymer by Solvent Extraction Method

The homopolymer (PAN) was separated from the crude graft copolymer by carrying out exhaustive Soxhlet extraction with DMF. After the complete removal of thehomopolymer, the pure graft copolymer was dried at 40 °C under vacuum until a constant weight was obtained.

### 4.3. Isolation of Grafted Chains 

The graft copolymer of Na–PCMSA containing PAN was hydrolyzed by refluxing it for 12 h in 1N HCl [43]. After all the Na–PCMSA went into the solution, a resinous mass was obtained, which was characterized with FTIR spectroscopy.

### 4.4. Alkaline Hydrolysis

Methanol precipitation method [40] was used to form the hydrogel by alkaline hydrolysis of the optimally synthesized graft copolymer, Na–PCMSA–g–PAN (%G = 316.53, %GE = 99.31). 10.0 g of the Na–PCMSA–g–PAN was dispersed in 100 mL 0.7N sodium hydroxide solution and gently stirred in the base under atmospheric conditions (5 min). The saponification was carried out by heating the dispersion at 90–95°C with occasional stirring until the color of the mixture changed from deep orange-red to light yellow (~2.5 h). The pasty mixture was cooled to room temperature, and methanol (5 × 10 mL) was added carefully to the gelled product while mixing. To completethe precipitation of the hydrogel, 200 mL additional methanol was added after 1 h to the yellow dispersion of the hydrogel (H–Na–PCMSA–g–PAN). The supernatant wasdecanted after 30 min, and 300 mL fresh methanol was then further added and kept for 24 h to remove water completely. The hydrogel obtained in powder form was thoroughly washed with fresh methanol, finally dried at 50 °C, and stored in a vacuum desiccator.

### 4.5. Characterization Methods

#### 4.5.1. FTIR Spectroscopy

The FTIR spectra of Na–PCMSA ($\bar{\mathrm{DS}}$ = 1.10), Na–PCMSA–g–PAN (%G = 316.53), PAN, and the superabsorbent hydrogel H–Na–PCMSA–g–PAN were recorded via KBr pellet method using Nicolet Impact 400D Fourier Transform Infrared Spectrophotometer between 400 and 4000 cm^−1^.

#### 4.5.2. Thermogravimetric Analysis (TGA)

The TGA traces of Na–PCMSA ($\bar{\mathrm{DS}}$ = 1.10), Na–PCMSA–g–PAN (%G = 316.53), and the superabsorbent hydrogel H–Na–PCMSA–g–PAN were obtained from Perkin Elmer Pyris 1 TGA, STA 8000, in an inert atmosphere (nitrogen) at a heating rate of 10 °C/min.

#### 4.5.3. Scanning Electron Microscopy (SEM)

Surface morphology of Na–PCMSA ($\bar{\mathrm{DS}}$ = 1.10), Na–PCMSA–g–PAN (%G = 316.53), and the superabsorbent hydrogel, H–Na–PCMSA–g–PAN, were analyzed with the help of ESEM TMP + EDAX, Philips make model.

## Figures and Tables

**Table 1:** Thermogravimetric data of Na–PCMSA ($\bar{DS}$ = 1.10), Na–PCMSA–g–PAN (%G = 316.53), and the superabsorbent hydrogel H–Na–PCMSA–g–PAN samples.

Sample	IDT	FDT	T_10_	T_50_	Temperature (°C) at Weight Loss			Temperature Range	T_max_	Weight Loss	Char Yield at 800 °C
	(°C)	(°C)	(°C)	(°C)	20%	40%	60%	(°C)	(°C)	(°C)	(%)
S_1_	140	796.53	138.14	271.17	191	219	459	50–100	--	7.19	19.28
								135.95–280.85	212.88 (34.52) ^a^	40.64	
								552.40–795.81	701.51 (71.12) ^a^	19.35	
S_2_	140	798.72	215.99	588.03	274.46	482.04	683.05	50–100	--	2.09	30.88
								66.88–136.13	92.82 (2.15) ^a^	2.20	
								136.13–239.93	211.79 (9.27) ^a^	9.30	
								239.93–317.77	270.26 (18.95) ^a^	11.88	
								341.53–430.15	384.83 (31.80) ^a^	8.47	
								501.60–776.24	592.42 (50.43) ^a^	25.56	
S_3_	97.71	797.81	87.35	488.62	234.44	387.03	604.29	50–100	--	9.07	20.42
								154.41–263.50	233.35 (20.26) ^a^	9.33	
								263.50–469.99	378.25 (38.41) ^a^	24.21	
								530.65–782.64	611.79 (61.79) ^a^	25.93	

^a^ the values correspond to respective weight loss, S_1_ = Na–PCMSA; S_2_ = Na–PCMSA–g–PAN; S_3_ = H–Na–PCMSA–g–PAN.

## Data Availability

Not applicable.
